# Association of bullous pemphigoïd and squamous cell carcinoma of the lip: a simple coïncidence?

**DOI:** 10.11604/pamj.2015.20.396.6804

**Published:** 2015-04-22

**Authors:** Hind Benhiba, Badredine Hassam

**Affiliations:** 1 Department of Dermatology, Ibn Sina Hospital, Faculty of Medecine and Pharmacy, University Mohamed V- Souissi, Rabat, Morocco

**Keywords:** Bullous pemphigoid, squamous cell carcinoma, lip

## Image in medicine

A 64-year-old smoking male presented with an abrupt onset of pruritic erythematous plaques predominantly in a sternal distribution for 2 weeks. Subsequently, a crop of tense vesicles and bullae appeared over these lesions. Physical examination revealed bullous lesions localized on the sternal region. Nikolsky sign and bulla spread sign were negative. Also, the patient presented ulcerated lesion of the midportion of lower lip. The histology of lip biopsy showed squamous cell carcinoma. The general investigations didn't reveal any metastasis. Besides, Histopathological examination of bullous lesions revealed a sub-epidermal blister with abundant fibrin, neutrophils, and eosinophils in the blister cavity suggestive of BP. Direct immunofluorescence revealed linear deposits of IgG and C3 along the basement membrane zone without intercellular deposits. Salt split skin technique by direct immunofluorescence, showed immunoreactants on both, epidermal and dermal sides of the split confirming BP as the diagnosis. The BP was treated by dermo-steroids and the patient was refered for oncological surgery and radiotherapy for the squamous cell carcinoma of the lip. The association of BP with cutaneous malignancies has always been a matter of debate with no consensus reached. Despite many published case reports and trials, a definite association is lacking. The time of presentation of malignancy can be variable, with malignancy presenting either before, concurrently or after the appearance of BP. BP has been reported with various malignancies especially of the gastrointestinal tract. To the best of our knowledge, this is the first report of BP associated with squamous cell carcinoma of the lower lip.

**Figure 1:**
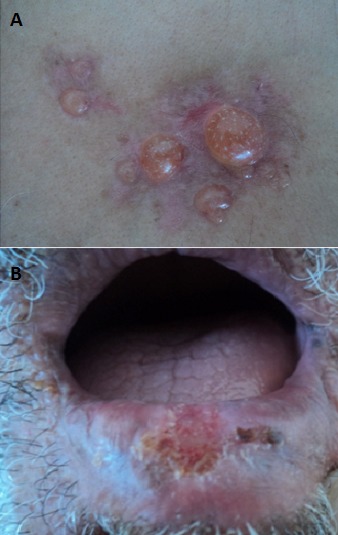
A) bullous lesions localized on the sternal region; B) ulcerated lesion of the midportion of lower lip

